# Anti-inflammatory effects of PGE_2_ in the lung: role of the EP_4_ receptor subtype

**DOI:** 10.1136/thoraxjnl-2014-206592

**Published:** 2015-05-04

**Authors:** Mark A Birrell, Sarah A Maher, Bilel Dekkak, Victoria Jones, Sissie Wong, Peter Brook, Maria G Belvisi

**Affiliations:** 1 Faculty of Medicine, Department of Respiratory Pharmacology, National Heart and Lung Institute, Imperial College London, London, UK; 2 MRC-Asthma UK Centre in Allergic Mechanisms of Asthma

**Keywords:** Asthma Pharmacology, Asthma Mechanisms, COPD Pharmacology

## Abstract

**Background:**

Asthma and chronic obstructive pulmonary disease (COPD) are chronic inflammatory diseases of the airway. Current treatment options (long acting β-adrenoceptor agonists and glucocorticosteroids) are not optimal as they are only effective in certain patient groups and safety concerns exist regarding both compound classes. Therefore, novel bronchodilator and anti-inflammatory strategies are being pursued. Prostaglandin E_2_ (PGE_2_) is an arachidonic acid-derived eicosanoid produced by the lung which acts on four different G-protein coupled receptors (EP_1–4_) to cause an array of beneficial and deleterious effects. The aim of this study was to identify the EP receptor mediating the anti-inflammatory actions of PGE_2_ in the lung using a range of cell-based assays and in vivo models.

**Methods and results:**

It was demonstrated in three distinct model systems (innate stimulus, lipopolysaccharide (LPS); allergic response, ovalbumin (OVA); inhaled pollutant, cigarette smoke) that mice missing functional EP_4_ (*Ptger4*^−/−^) receptors had higher levels of airway inflammation, suggesting that endogenous PGE_2_ was suppressing inflammation via EP_4_ receptor activation. Cell-based assay systems (murine and human monocytes/alveolar macrophages) demonstrated that PGE_2_ inhibited cytokine release from LPS-stimulated cells and that this was mimicked by an EP_4_ (but not EP_1–3_) receptor agonist and inhibited by an EP_4_ receptor antagonist. The anti-inflammatory effect occurred at the transcriptional level and was via the adenylyl cyclase/cAMP/ cAMP-dependent protein kinase (PKA) axis.

**Conclusion:**

This study demonstrates that EP_4_ receptor activation is responsible for the anti-inflammatory activity of PGE_2_ in a range of disease relevant models and, as such, could represent a novel therapeutic target for chronic airway inflammatory conditions.

**Key messages**

**What is the key question?**

- Prostaglandin E_2_ (PGE_2_) possesses anti-inflammatory and bronchodilator activity in clinical studies but unfortunately this is accompanied by airway irritancy, so identification of the receptor mediating the beneficial effects could lead to the development of a novel therapeutic for chronic inflammatory diseases characterised by airflow obstruction.

**What is the bottom line?**

- EP_4_ receptor activation is responsible for the anti-inflammatory activity of PGE_2_ across a range of inflammatory in vivo models and in human cell-based assays.

**Why read on?**

- This study identifies for the first time in a range of clinically relevant models the EP_4_ receptor and its associated signalling pathway as a target for a dual bronchodilator/anti-inflammatory therapy for the treatment of respiratory diseases such as asthma and chronic obstructive pulmonary disease.

## Introduction

Asthma and chronic obstructive pulmonary disease (COPD) are respiratory diseases with an increasing global prevalence that represent a social and economic burden for both industrialised and developing countries. The World Health Organization states that the number of patients with asthma is 300 million and predicts that this figure will rise to 400 million by 2025,^1^ while there are 600 million people with COPD worldwide and the disease is predicted to be the third ranked leading cause of death by 2020.^1^ Exacerbations are common events in the lives of patients with asthma and COPD. These episodes are often associated with infections by viruses or bacteria and cause worsening of symptoms, which can be fatal. Often these heightened symptoms are far less responsive to normal treatments and are associated with increased healthcare costs and societal impact. There is therefore an urgent need to develop safe and effective therapies for these respiratory diseases.

It has been recognised for many years that prostaglandin E_2_ (PGE_2_) is produced by many cell types within the lung.^2^ Furthermore, exogenous PGE_2_ has been shown to have bronchodilator and anti-inflammatory properties in rodent models^3^ and human subjects.^4^ ^5^ Thus, PGE_2_ could represent an effective treatment, but it is associated with airway irritation and coughing^6^ and has a very short half-life and, as such, has an inappropriate pharmacokinetic profile for general clinical use.

The biological actions of PGE_2_ are predominantly mediated by the activation of the four EP receptors, EP_1–4_.^7^ We and others have recently shown that the bronchodilator properties of PGE_2_ in human airways are mediated via activation of the EP_4_ receptor,^8^ whereas the undesirable triggering of airway sensory nerves appears to be via its activity at the EP_3_ receptor.^9^ However, it is still not clear which receptor is involved in mediating the anti-inflammatory effects of PGE_2_. Identification of a receptor mediating the anti-inflammatory and bronchodilator effects of PGE_2_ without the airway irritant activity would harness the beneficial effects while avoiding the undesirable properties.

To achieve this aim we profiled the inflammatory status of EP receptor knockout (KO) mice in an array of preclinical respiratory disease model systems (endotoxin: innate; allergen: asthma-like; and smoke: COPD-like). To investigate the signalling mechanisms involved, we used in vitro murine and human cell-based assay systems. In vitro assays confirmed the in vivo data which suggested that EP_4_ receptor activation is responsible for the anti-inflammatory activity of PGE_2_ and that the effect was at the level of cytokine gene transcription via an adenylyl cyclase (AC)/cAMP/cAMP-dependent protein kinase (PKA) axis.

## Materials and methods

### Role of EP receptors in murine respiratory models

Breeding pairs of C57bl/6 wild type and EP receptor gene (*Ptger1–Ptger4*) KO mice were purchased from Harlan (Bicester, Oxon, UK) and provided by Dr Shuh Narumiya, Kyoto University, respectively. *Ptger1–Ptger3* KO mice were backcrossed on the C57bl/6 at least eight times. *Ptger4*−/− mice do not survive on the C57bl/6 background^10^ and were backcrossed on a mixed background of 129/Ola×C57bl/6. Corresponding wild type colonies were generated. Male mice (18–20 g) were generated from established breeding colonies maintained internally. Experiments were performed under Home Office project licence (PPL 70/7212) and procedures adhered to the Animals (Scientific Procedures) Act 1986 and according to the ARRIVE guidelines.^11^

Mice were challenged with inhaled lipopolysaccharide (LPS) (innate response), ovalbumin (OVA) (allergic: asthma-like) or cigarette smoke as previously described.^12–14^ Briefly, for the innate response model, mice were challenged with an aerosol of endotoxin-free saline (for 30 min) or LPS (1 mg/mL, *Escherichia coli* serotype 0111:B4). For the asthma model, mice were sensitised with OVA (intraperitoneally with alum on days 0 and 14) and on days 24, 25 and 26 mice were intranasally challenged with saline or OVA (50 µg in 50 µL) under isoflurane anaesthesia. For the COPD model, mice were challenged with cigarette smoke or room air for 1 h twice a day for 3 days. Airway cellular inflammation was assessed via differential counting in the lavage and EP mRNA receptor expression determined in the lung tissue (over time at 2, 6, 24 and 48 h after final challenge).

### Role of EP receptors in cell-based systems

Cultured murine (J774) and human (THP-1) monocytes (treated with the cyclo-oxygenase inhibitor indomethacin) were challenged with LPS in the presence and absence of PGE_2._ Cytokine levels in the culture medium were assessed using ELISA. In parallel experiments, the impact of specific EP receptor agonists EP_1_ (ONO-D1-004), EP_2_ (ONO-AE1-259), EP_3_ (ONO-AE-248), EP_4_ (ONO-AE1-329) and antagonists EP_2_ (PF-04418948) and EP_4_ (ONO-AE3-208) were studied. Concentrations used were selected from previous studies and published literature.^8^ ^9^ ^15^ Dimethyl sulfoxide was used as the vehicle, final concentration 0.001–2% (v/v). EP receptor expression was determined in THP-1 cells and primary human alveolar macrophages by RT-PCR.

Cytokine production was measured at the mRNA (RT-PCR 2 h post-challenge) and protein (ELISA 22 h post-challenge) level to assess the effect of EP_4_ agonist on transcription/translation. To further explore the signalling mechanism, the role of AC (AC activator: forskolin; inactive control: 1,9 dideoxyforskolin) and PKA/exchange proteins activated by cAMP (EPAC) (PKA activator: 6-Bnz-cAMP; EPAC activator: SP-8-pCPT-2′-O-Me-cAMP; and PKA inhibitor: RP-8-CPT-cAMPS) were explored; concentrations were selected from published data and unpublished concentration–response curves.^16^ ^17^ The key experiment was repeated in primary human alveolar macrophages as previously described.^18^ Human lung tissue was purchased from the International Institute for the Advancement of Medicine (Edison, New Jersey, USA). In all cases, consent for use of the tissue in scientific research and ethics approval were obtained from the Royal Brompton and Harefield Trust.

### Data analysis and statistics

Data are presented as mean±SEM and statistical significance was assumed when p<0.05. N numbers and statistical tests used were the Mann–Whitney test when two datasets were compared and Kruskal–Wallis ANOVA followed by Dunn’s multiple comparison for three or more datasets. The specific test used is indicated in the figure legends.

## Results

### Role of EP receptors in murine respiratory models of airway inflammation

**Figure 1:**
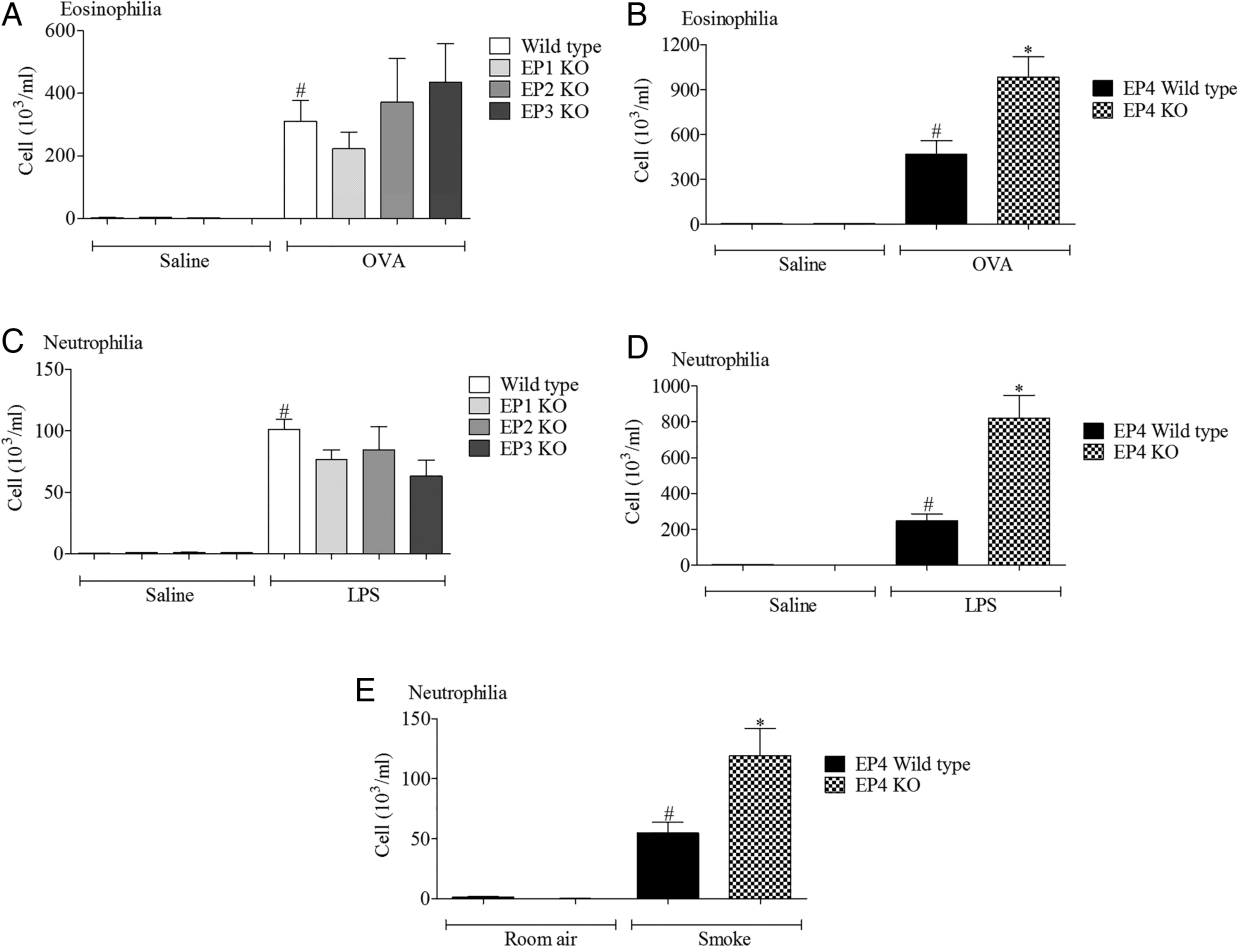
Role of EP receptors in murine respiratory models. (A, B) Eosinophils in lavage fluid from mice sensitised and challenged with ovalbumin (OVA), wild type controls versus EP_1–3_ knockout (KO) mice (A) or wild type controls versus EP_4_ KO mice (B). (C, D) Neutrophils in lavage fluid from mice challenged with lipopolysaccharide (LPS), wild type controls versus EP_1–3_ KO mice (C) or wild type controls versus EP_4_ KO mice (D). (E) Neutrophils in lavage fluid from mice exposed to cigarette smoke, wild type controls versus EP_4_ KO mice. Data shown are mean±SEM (n=8). #Statistical significance between control challenge and stimulation (p<0.05, Mann–Whitney test). *Statistical significance between wild type and KO mice (p<0.05, Mann–Whitney test).

Wild type mice challenged with the various disease relevant stimuli evoked the expected inflammatory responses associated with increases in a particular cell type. In particular, increases in airway neutrophils were observed in the LPS and cigarette smoke models and eosinophils after antigen challenge (figure 1). In the LPS and allergen models, inflammatory cell infiltration was significantly increased in the EP_4_ receptor (*Ptger4*−/−) KO mice (with no change in EP_1–3_ KO mice) compared with the wild type control, suggesting a protective role for EP_4_ receptor activation (figure 1). To determine if this phenomenon was restricted to these inflammatory stimuli, we compared inflammatory cell infiltration into the airway following cigarette smoke challenge and found that absence of the EP_4_ receptor appeared to enhance the inflammatory response (figure 1E).

The EP_4_ KO mice, of necessity, were on a different genetic background to the other three KO lines.^10^ This difference in genetic background between the studies with EP_1–3_ and EP_4_ receptor KO mice could conceivably complicate the data interpretation. Thus, in order to provide evidence that functional changes were associated with the ‘disease phenotype’ and not specific to the genetic background of the mice, we assessed EP receptor expression in the allergen and LPS models in mice bred on a pure C57bl/6 background. While we could not detect a robust temporal change in the levels of EP_1–3_ receptor mRNA, EP_4_ receptor mRNA levels were significantly increased across a number of time points in both inflammatory models (see online supplementary figures S1 and S2).

### Anti-inflammatory role of EP receptors in cell-based assay systems

**Figure 2:**
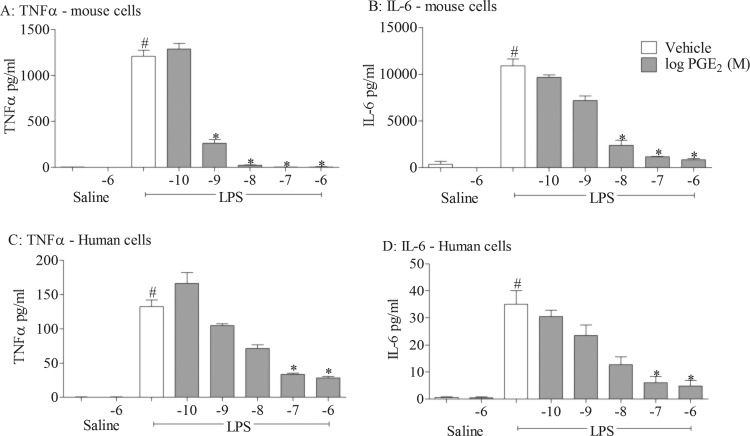
Effect of prostaglandin E_2_ (PGE_2_) on cytokine release in cell-based assays. Effect of increasing concentrations of PGE_2_ on levels of tumour necrosis factor α (TNFα) (A, C) and interleukin 6 (IL-6) (B, D) in culture medium from mouse (J774; A, B) and human (THP-1; C, D) THP-1 cells stimulated with vehicle (saline) or lipopolysaccharide (LPS; 0.1 μg/mL). Data shown are mean±SEM of three experimental runs performed in duplicate. #Statistical significance between vehicle and stimulation (p<0.05, Mann–Whitney test). *Statistical significance between stimulation and PGE_2_-treated cells (p<0.05, Kruskal–Wallis ANOVA followed by Dunn’s multiple comparison test).

**Figure 3:**
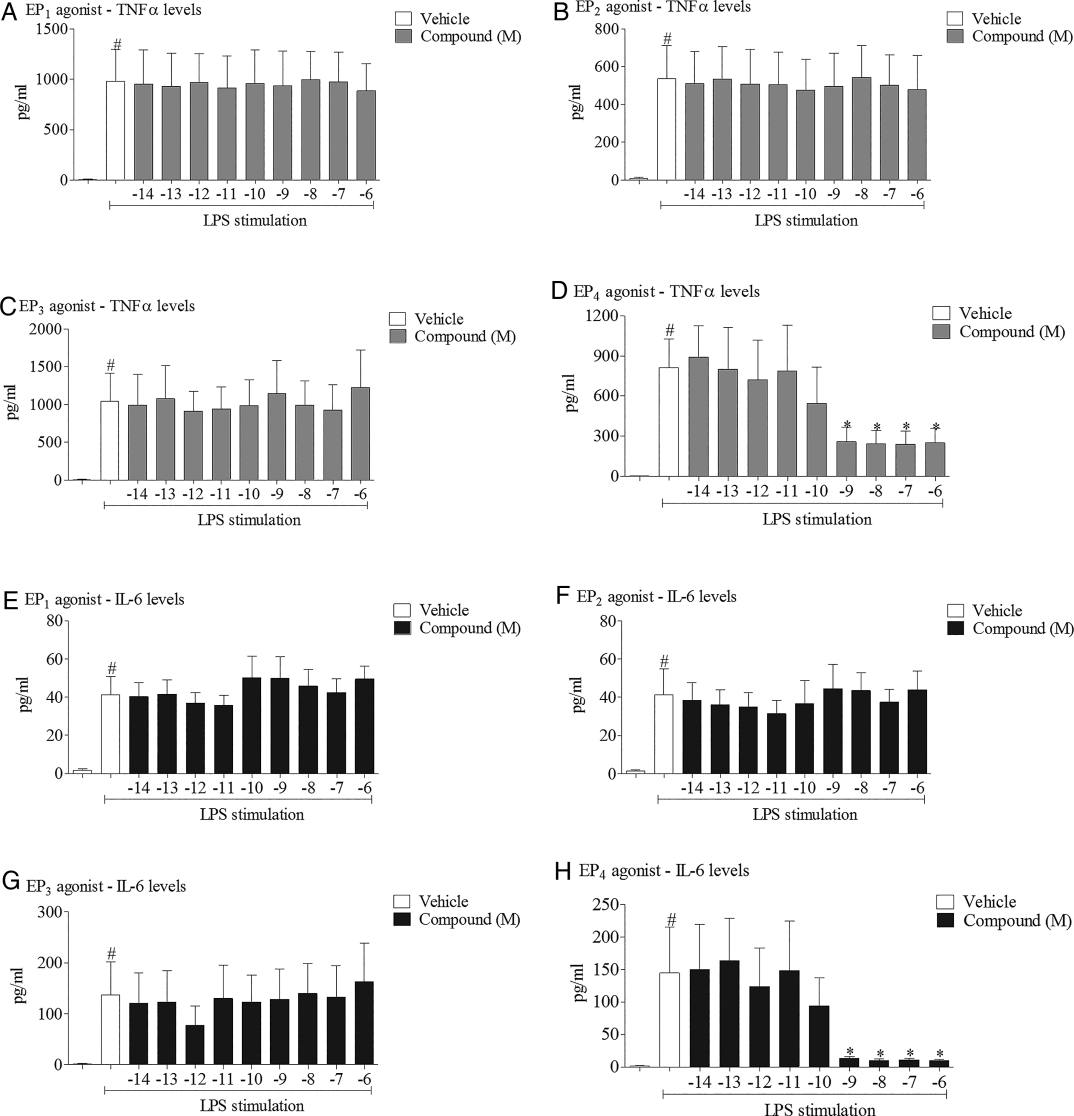
Effect of selective EP receptor agonists on cytokine release. Effect of increasing concentrations of EP receptor agonists (EP_1_: ONO-D1-004, EP_2_: ONO-AE1-259, EP_3_: ONO-AE-248, EP_4_: ONO-AE1-329) on levels of tumour necrosis factor α (TNFα) (A–D) and interleukin 6 (IL-6) (E–H) in culture medium from human (THP-1) monocytes stimulated with vehicle (saline) or lipopolysaccharide (LPS; 0.1 μg/mL). Data shown are mean±SEM of three experimental runs performed in duplicate. #Statistical significance between vehicle and stimulation (p<0.05, Mann–Whitney test). *statistical significance between stimulation and agonist treated cells (p<0.05, Kruskal–Wallis ANOVA followed by Dunn’s multiple comparison test).

In three different disease relevant in vivo models of airway inflammation we found that the inflammatory response was increased in EP_4_ receptor KO mice, suggesting an anti-inflammatory role for endogenous prostaglandins via activation of EP_4_ receptors. In order to perform more in-depth investigations using pharmacological tools without the appropriate pharmacokinetic profile for in vivo studies and in order to provide translational data, we performed studies in human cell-based assays. To investigate the anti-inflammatory potential of PGE_2_, we examined the effect of exogenously added PGE_2_ on inflammatory responses to one of the stimuli used in the murine model, namely LPS. All experiments were performed in the presence of a broad spectrum cyclo-oxygenase inhibitor to block endogenous prostanoid production which may complicate data interpretation. PGE_2_ produced a concentration-related inhibition of LPS-induced cytokine production in mouse and human monocytes (figure 2). To investigate which receptor(s) were central to the anti-inflammatory effect seen with PGE_2_, we repeated the study using selective EP agonists and found that only the EP_4_ receptor agonist (ONO-AE1-329) modulated cytokine levels at concentrations commensurate with the reported nanomolar potency of this ligand at the EP_4_ receptor (figure 3D, H). In a parallel experiment, the same EP receptor agonists did not trigger cytokine release under non-stimulated conditions (see online supplementary figure S3). Interestingly, EP_4_ receptor mRNA expression was the predominant EP receptor subtype expressed at the mRNA level in these cells and primary human alveolar macrophages (see online supplementary figure S4). These data suggest that the EP receptor expression profile and the predominance of EP_4_ explains why the anti-inflammatory activity of PGE_2_ is mediated via the EP_4_ receptor.

**Figure 4:**
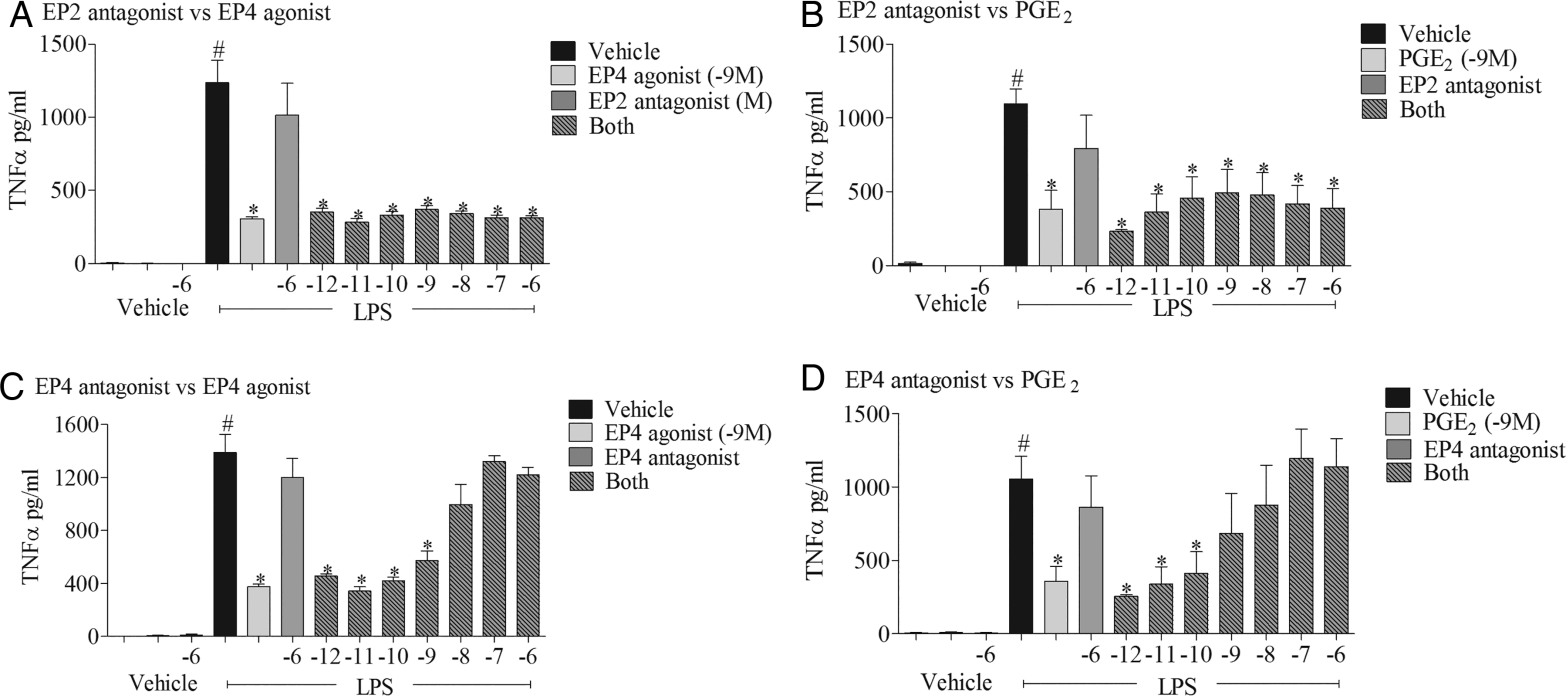
Effect of selective EP receptor antagonists on inhibitory effect of prostaglandin E_2_ (PGE_2_) and EP_4_ agonist-induced cytokine release. Effect of EP_4_ receptor agonist (ONO-AE1-329, 1 nM) (A, C) or PGE_2_ (1 nM) (B, D) in the presence of increasing concentrations of EP_2_ (PF-04418948) or EP_4_ (ONO-AE3-208) receptor antagonists on tumour necrosis factor α (TNFα) levels in culture medium from human (THP-1) monocytes stimulated with vehicle (saline) or lipopolysaccharide (LPS; 0.1 μg/mL). Data shown are mean±SEM of three experimental runs performed in duplicate. #Statistical significance between vehicle and stimulation (p<0.05, Mann–Whitney test). *Statistical significance between stimulation and agonist treated cells (p<0.05, Kruskal–Wallis ANOVA followed by Dunn’s multiple comparison test).

To confirm that the EP_4_ receptor is central to the anti-inflammatory properties of PGE_2_, we performed experiments in the presence of selective EP_2_ and EP_4_ receptor antagonists (PF-04418948 and ONO-AE3-208, respectively) and focused on tumour necrosis factor α (TNFα) production as an example cytokine. The data clearly show that the EP_4_ receptor antagonist blocks the anti-inflammatory activity of both the selective EP_4_ receptor agonist and PGE_2_ itself (figure 4C, D), with no effect of the EP_2_ receptor antagonist.

**Figure 5:**
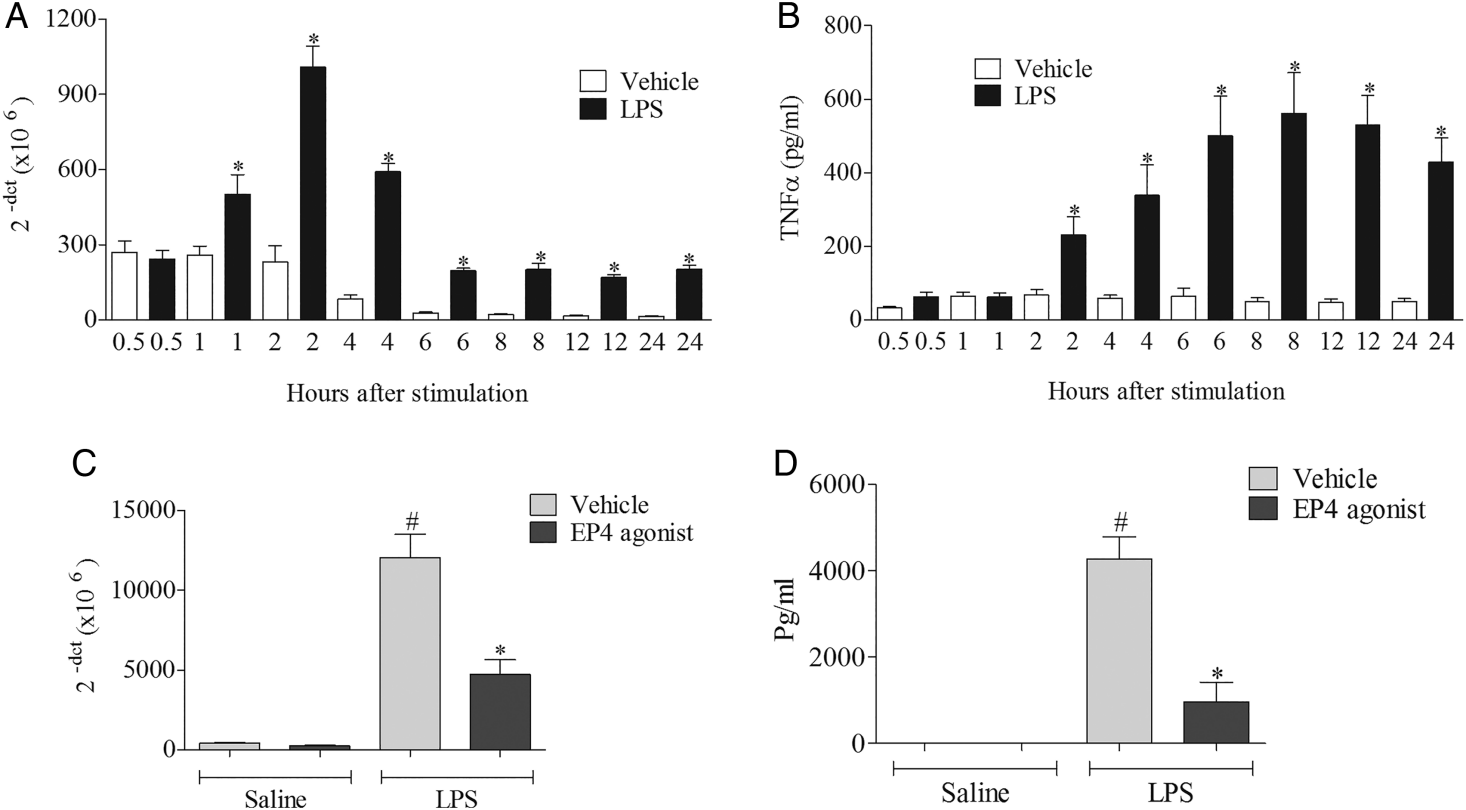
Assessing the effect of EP_4_ receptor activation on transcription and translation of tumour necrosis factor α (TNFα). TNFα mRNA (A) and protein production (B) following treatment with vehicle (saline) or lipopolysaccharide (LPS; 0.1 μg/mL) over time in the culture medium of human (THP-1) monocytes. (C) TNFα mRNA measured 2 h post stimulation and (D) protein measured 22 h post stimulation in the presence of EP_4_ receptor agonist (ONO-AE1-329). Data shown are mean±SEM of three experimental runs performed in duplicate. #Statistical significance between vehicle and stimulation (p<0.05, Mann–Whitney test). *Statistical significance between stimulation and agonist treated cells (p<0.05, Mann–Whitney test).

In order to investigate whether the production of TNFα was regulated at the transcriptional or translational level, we had to determine a suitable time point to measure mRNA production. The 2 h time point was selected to measure mRNA and protein production was assessed 22 h after stimulation (figure 5A). The EP_4_ receptor agonist modulated both the mRNA and protein levels, which suggests that activation of the receptor modulates cytokine production at the transcriptional level (figure 5C, D).

**Figure 6:**
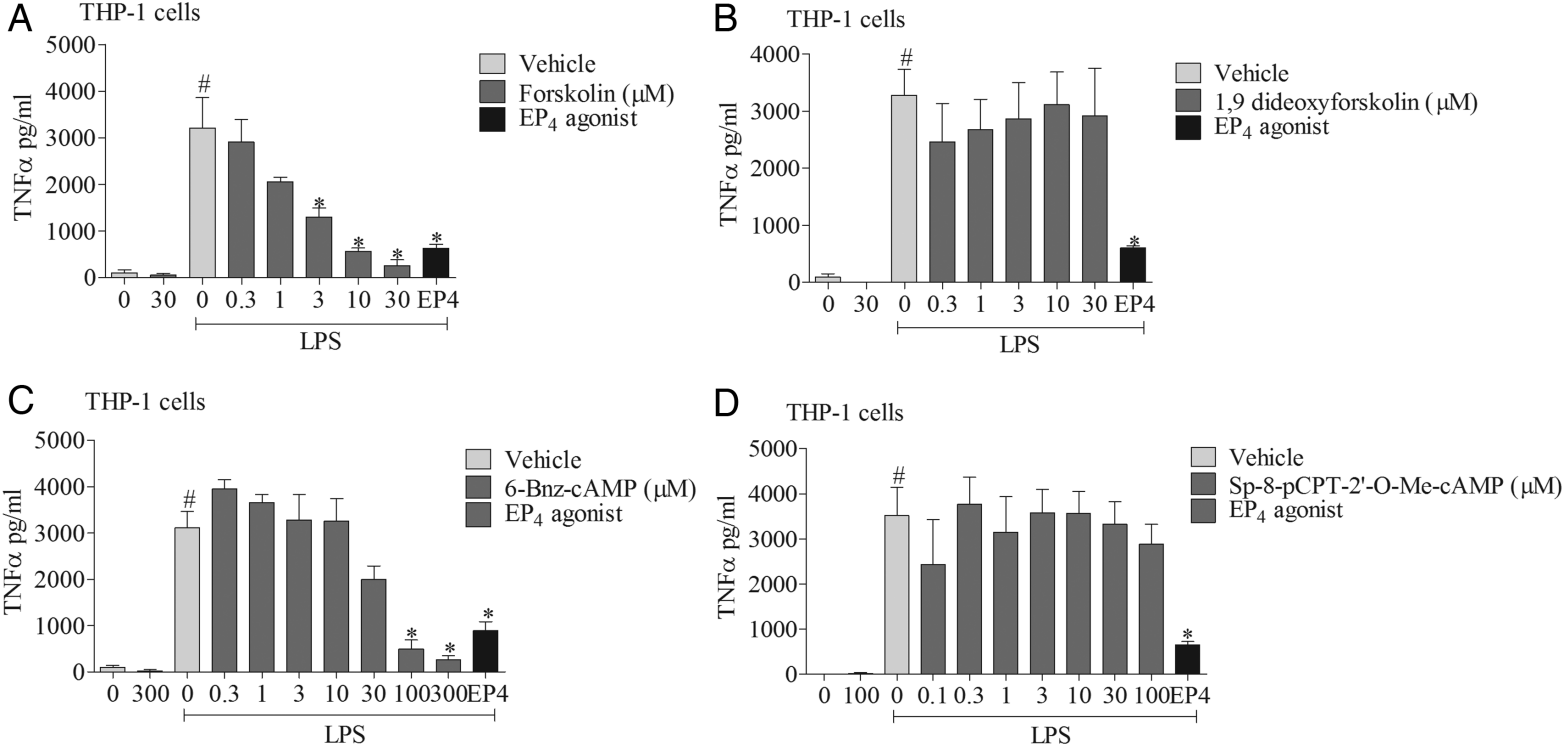
EP_4_ receptor activation and downstream signalling pathways. The effect of vehicle, adenylyl cyclase (AC) activator (A), AC activator negative control (B), cAMP-dependent protein kinase (PKA) activator (C), or exchange proteins activated by cAMP (EPAC) activator (D) on tumour necrosis factor α (TNFα) release from human (THP-1) monocytes stimulated with vehicle (saline) or lipopolysaccharide (LPS; 0.1 μg/mL). The EP_4_ receptor agonist (ONO-AE1-329, 1 nM) was included as a positive control. Data shown are mean±SEM of three experimental runs performed in duplicate. #Statistical significance between vehicle and stimulation (p<0.05, Mann–Whitney test). *Statistical significance between stimulation and agonist treated cells (p<0.05, Kruskal–Wallis ANOVA followed by Dunn’s multiple comparison test).

**Figure 7:**
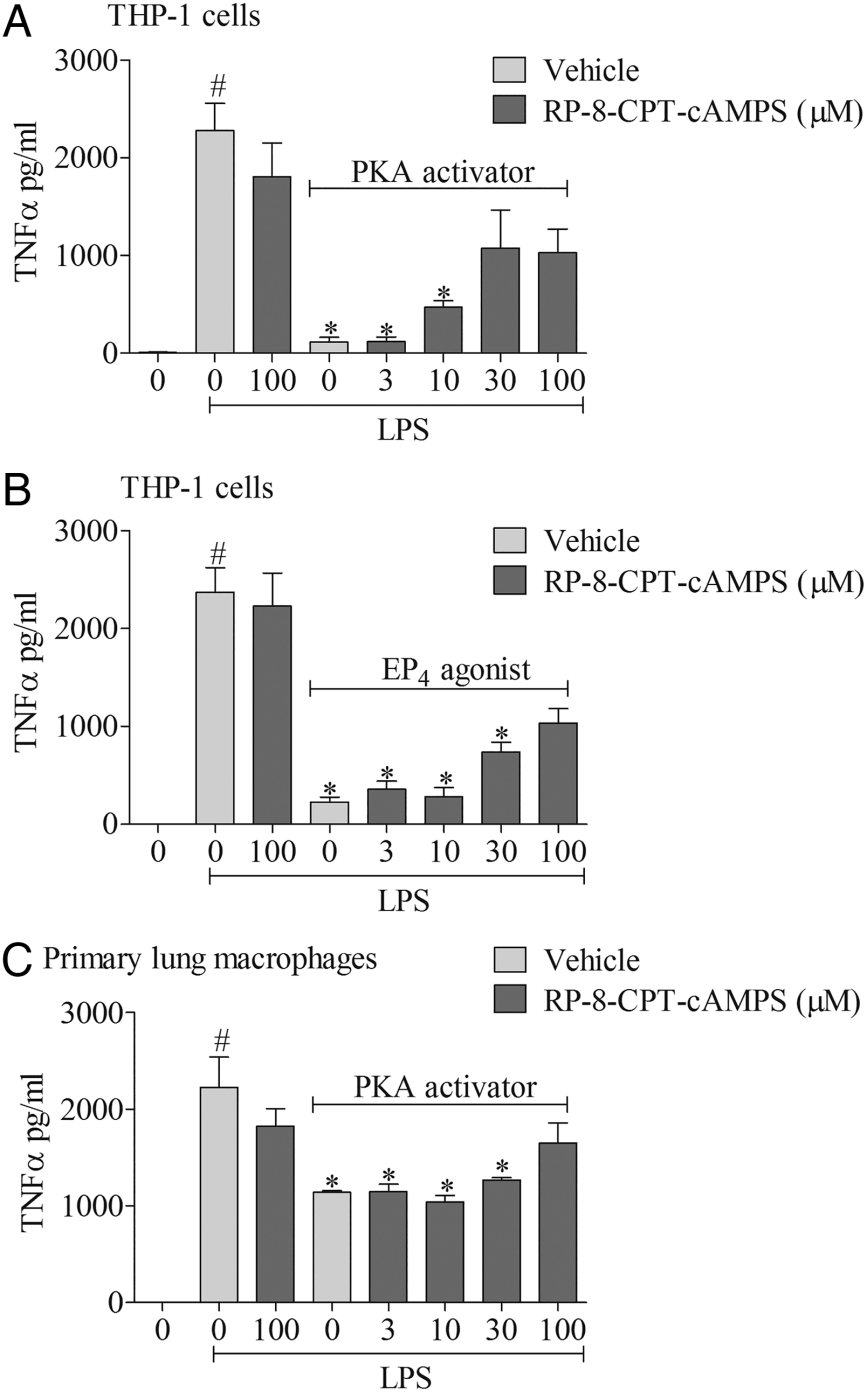
EP_4_ receptor activation and downstream signalling pathways. Effect of a cAMP-dependent protein kinase (PKA) activator (6-Bnz-cAMP, 100 μM) plus increasing concentrations of a PKA inhibitor (RP-8-CPT-cAMPs) (A) or EP_4_ receptor agonist (ONO-AE1-329, 1 nM) plus the PKA inhibitor (B). PKA activator plus increasing concentrations of PKA inhibitor on tumour necrosis factor α (TNFα) levels in culture medium from human (THP-1) monocytes stimulated with vehicle (saline) or lipopolysaccharide (LPS; 0.1 μg/mL). The effect of a PKA activator plus increasing concentrations of PKA inhibitor (C) in primary human alveolar macrophages stimulated with LPS or vehicle. Data shown are mean±SEM of three experimental runs performed in duplicate. #Statistical significance between vehicle and stimulation (p<0.05, Mann–Whitney test). *Statistical significance between stimulation and agonist treated cells (p<0.05, Kruskal–Wallis ANOVA followed by Dunn’s multiple comparison test).

To explore the signalling mechanisms involved, we investigated the effects of mimicking proposed EP_4_ receptor signalling involving the AC-PKA/EPAC pathways. The AC activator, but not its negative control (1,9 dideoxyforskolin, which shares many of the activities of forskolin but does not activate AC), caused a concentration-related inhibition of cytokine production which was of a similar magnitude to the EP_4_ receptor agonist (figure 6A, B). Similarly, an activator of PKA, but not EPAC, mirrored the effect of the EP_4_ receptor agonist (figure 6C, D). In order to determine if this proposed pathway was indeed triggered by activating the EP_4_ receptor, we employed a selective PKA blocker. The PKA inhibitor caused a concentration-related block of the anti-inflammatory properties of both a PKA activator and the EP_4_ receptor agonist (figure 7A, B). Finally, to demonstrate this pathway was active in primary cells, we repeated the observation in human alveolar macrophages (figure 7C).

## Discussion

Current anti-inflammatory therapy for asthma and COPD is focused on the use of glucocorticoids, but their efficacy is limited in COPD and in certain patient subgroups with asthma. Furthermore, there are significant patient compliance issues and safety concerns associated with their long-term use. Existing treatment of both diseases is focused on combination therapies which include a bronchodilator together with the glucocorticoid anti-inflammatory. Therefore, a single treatment with both anti-inflammatory and bronchodilator potential would be a preferred therapeutic option, especially if it could be developed as an oral medication to avoid compliance issues.

The beneficial properties of PGE_2_ in the lung have long been recognised, but irritancy issues and the poor pharmacokinetic profile of endogenous bioactive lipid mediators mean that these properties are yet to be fully harnessed. Previous studies have started to define the EP receptors associated with the beneficial and undesirable properties associated with PGE_2_ to identify a receptor subtype which could be targeted for the development of a therapeutic. For example, EP_4_ receptor activation has been proposed to be involved in PGE_2_-induced bronchodilation of human airway smooth muscle and EP_3_ receptor activation to trigger airway sensory nerves producing unwanted reflexes such as cough.^8^ ^9^ The aim of this study was to begin to explore which receptor(s) are required for the airway anti-inflammatory properties of PGE_2_.

The initial body of work was performed in three in vivo murine models with distinct phenotypes (modelling innate, allergic and COPD-like inflammation) and demonstrated that EP_4_ receptor KO mice had an enhanced cellular inflammation, suggesting an endogenous anti-inflammatory role for PGE_2_ acting on the EP_4_ receptor. Supporting these data was the observation that EP_4_ receptor gene expression was increased in the lungs after disease relevant challenges in standard (C57bl/6) wild type mice, suggesting that this observation was not specific to the genetic background of the mice used in the EP_4_ comparator studies. Consistent with these findings are the facts that most immune cell types have been reported to express EP_4_ receptors,^7^ ^19^ ^20^ and EP_4_ receptor activation reduces transendothelial migration of eosinophils and neutrophils^21^ ^22^ and induces suppression in a murine model of aspirin-triggered allergic hyperresponsiveness.^23^ In contrast to these observations, a protective role for EP_3_ receptor activation has been reported in a murine asthma model.^24^ Although a beneficial role for the EP_3_ receptor is not supported in studies demonstrating that PGE_2_—but not the EP_3_ receptor agonist sulprostone—exhibits anti-inflammatory effects in an allergic murine model, and in other studies suggesting that EP_3_ receptor activation is chemotactic for lung mast cells.^25^ Alternatively, other studies have reported that EP_2_ receptor activation inhibits asthma-related eosinophilic trafficking.^26^ ^27^ Although studies exist supporting a role for alternative receptors in mediating the anti-inflammatory effects of PGE_2_, often the study investigators have not had the opportunity to use the wide range of selective pharmacological tools and KO mice that we have been able to employ here. Furthermore, the majority of the published literature regarding the anti-inflammatory role of PGE_2_ is in agreement with our data suggesting a role for the EP_4_ receptor.

To provide further confirmatory data regarding the role of the EP_4_ receptor and to investigate the downstream signalling pathway, we used cell-based systems which are more amenable to study and where data interpretation is not limited by the pharmacokinetic limitations of tool compounds. This approach also enabled translation of findings in murine systems to human cells. Monocyte/macrophage cell types were used as the prevalent cell in the airway and the cell type most likely to have a common role in all three inflammatory responses that were modelled in the mouse. Furthermore, previous studies had demonstrated EP_4_ receptor gene expression in murine macrophages,^28^ the murine monocyte cell line J774 cells^29^ and human lung macrophages.^20^ ^30^ In these experiments, using one of the stimuli used in the model systems (endotoxin, LPS), PGE_2_ also had an anti-inflammatory role demonstrated as the inhibition of cytokine release via activation of the EP_4_ receptor. These data are consistent with previous studies that have reported a similar role for the EP_4_ receptor in a diverse range of cell-based assays.^19^ ^20^ ^29^ ^31^ ^32^ Interestingly, we did not observe an anti-inflammatory role for the EP_2_ receptor, as suggested by other studies. In addition, although we failed to note any pro-inflammatory effects of PGE_2_, it has been reported to increase levels of inflammatory mediators such as vascular endothelial growth factor through both EP_2_ and EP_4_ receptors.^33^

To investigate how activation of the EP_4_ receptor attenuates cytokine production, we probed the reported signalling mechanisms. EP_4_ receptors signal via activation of Gαs and AC and a subsequent increase in intracellular cAMP which can then activate PKA or EPAC.^7^ While others have suggested different signalling mechanisms such as EP_4_ receptor-associated protein (p105/NF-kB) and Gαi (PI3K/PKC),^32^ ^34^ ^35^ many report that the cAMP/PKA axis is central to the response.^7^ In our cell systems we demonstrated that the anti-inflammatory actions of PGE_2_ and the specific EP_4_ receptor agonist were mimicked by AC and PKA activators, but not an EPAC activator, and blocked by a PKA inhibitor. Furthermore, cytokine production was suppressed at the mRNA level, presumably by halting its transcription or by increasing mRNA degradation.

In conclusion, these findings present a convincing dataset that supports the hypothesis that the anti-inflammatory activity of PGE_2_ is mediated via activation of the EP_4_ receptor and that this mechanism is capable of suppressing airway inflammation that is triggered via an array of stimuli. We have shown that EP_4_ receptor KO mice have enhanced inflammation after innate, allergic and COPD-like conditions, and cytokine production from primary human alveolar macrophages is attenuated by an EP_4_ agonist in a cAMP/PKA-dependent manner. We suggest that an EP_4_ receptor agonist with an appropriate pharmacokinetic profile would harness the beneficial properties of PGE_2_ and make an effective anti-inflammatory and bronchodilator for the treatment of respiratory diseases such as asthma and COPD.

## Supplementary Material

Web figures
