# Supplementary material for: Anti-inflammatory effects of PGE2 in the lung: role of the EP4 receptor subtype
Source: Thorax. 2015 May 4;70(8):740–7. doi: 10.1136/thoraxjnl-2014-206592 (PMC4516010; doi:10.1136/thoraxjnl-2014-206592)

Supplementary Figure 1: EP receptor expression in the innate model

Panels depict the EP_{1-4} receptor mRNA levels in the lung tissue from mice challenged with aerosolised saline or LPS (1 mg/ml) for 30 minutes. Data shown is mean ± s.e.m., n = 8.

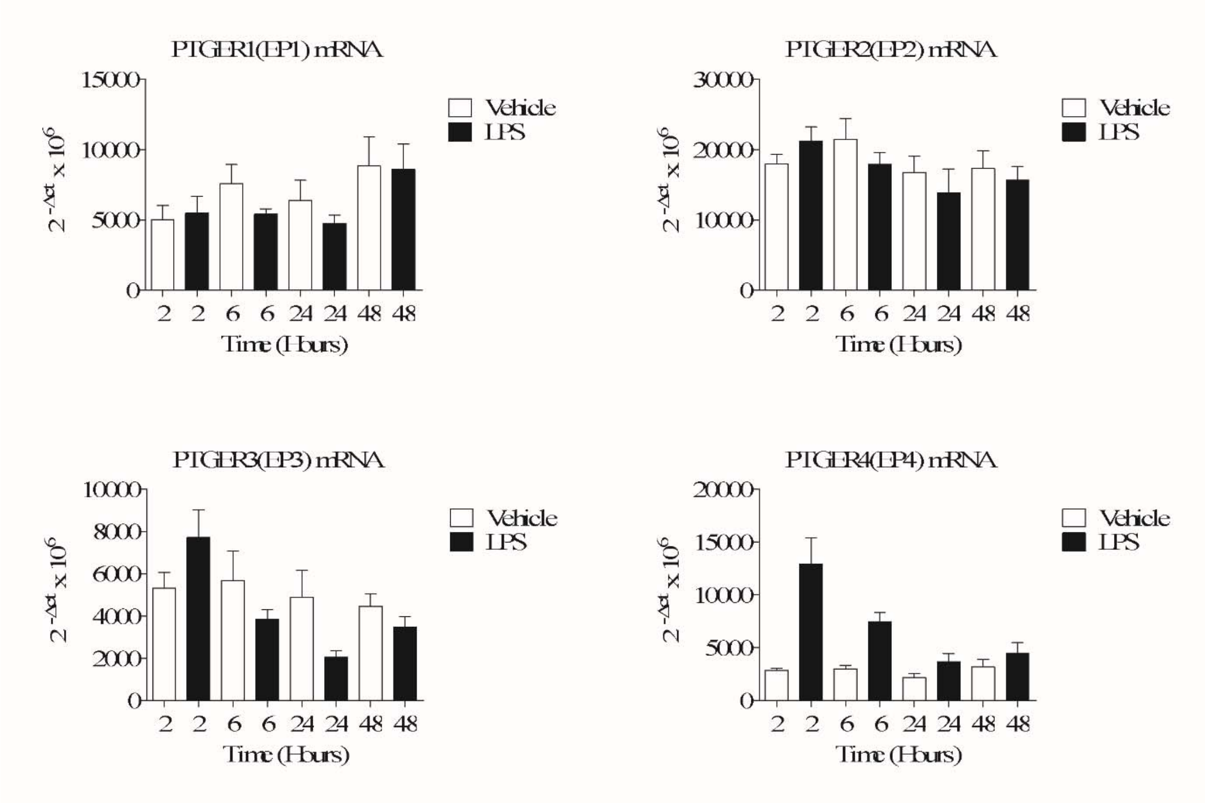

Supplementary Figure 2: EP receptor expression in the allergic model

Panels depict the EP_{1-4} receptor mRNA levels in lung tissue from sensitised mice challenged with intranasal saline or OVA once a day for 3 days. Data shown is mean ± s.e.m., n = 8.

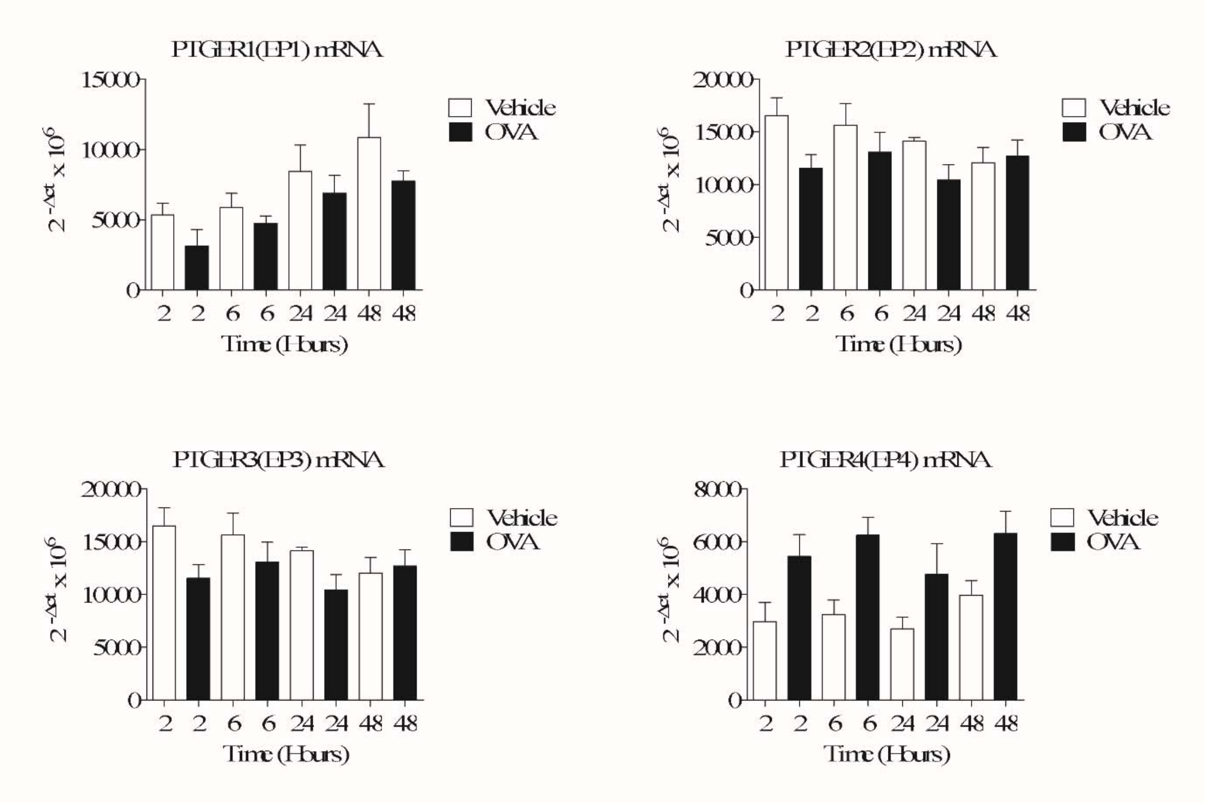

Supplementary Figure 3: Effect of selective EP receptor agonists on basal cytokine release

Effect of increasing concentrations of EP receptor agonists (EP_1: ONO-D1-004), EP_2: ONOAE1-259), EP_3: ONO-AE-248, EP_4: ONO-AE1-329 on TNF〈 (A-D) and IL-6 (E-H) levels in culture medium from unstimulated human (THP-1) monocytes. Data shown is mean ± s.e.m.

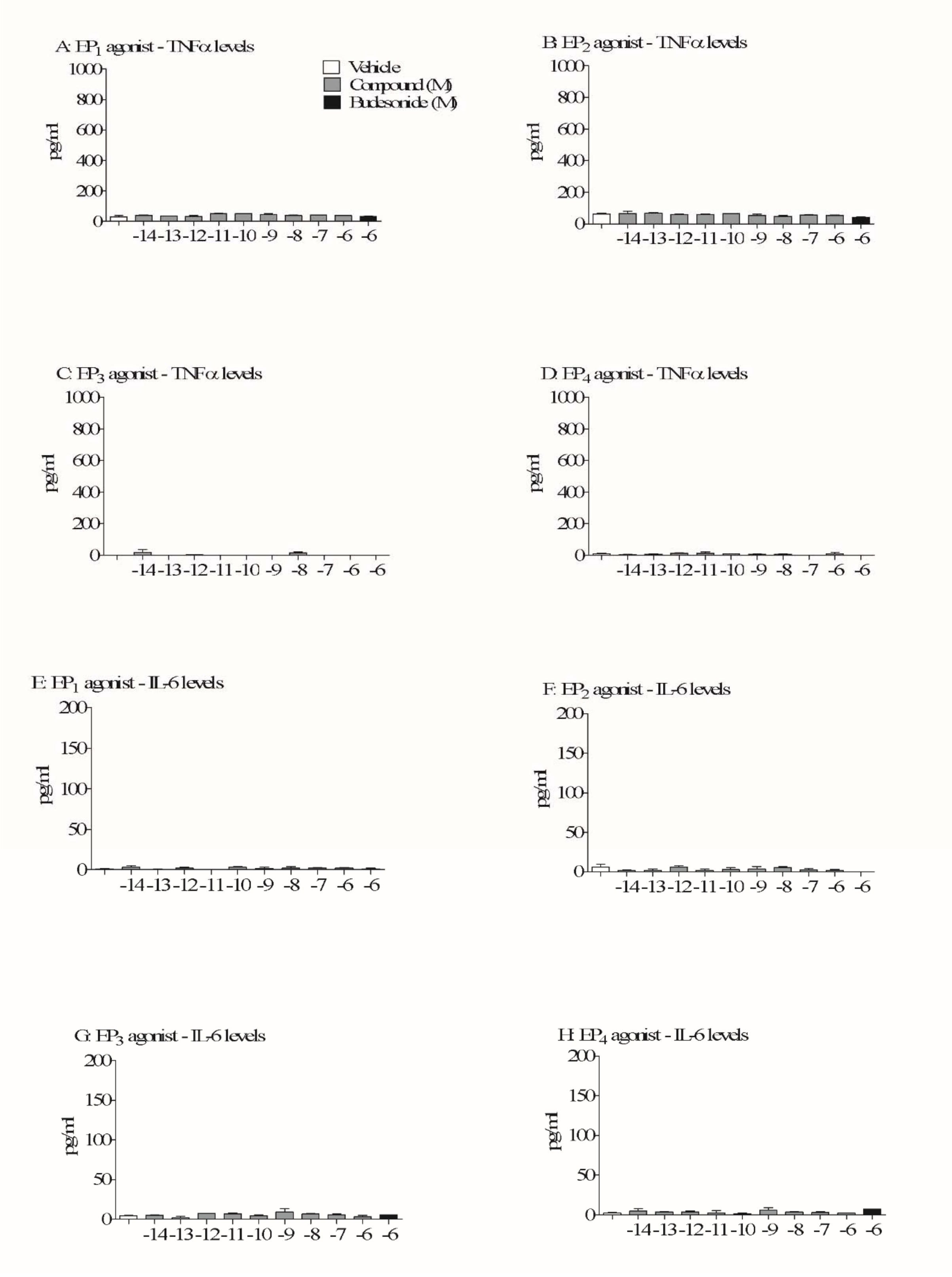

Supplementary Figure 4: EP receptor expression in human monocytes/macrophages

Panels depict the EP_{1-4} receptor mRNA levels in THP-1 cells (A) of human alveolar macrophages (B). Data shown is mean ± s.e.m., n = 3-6.

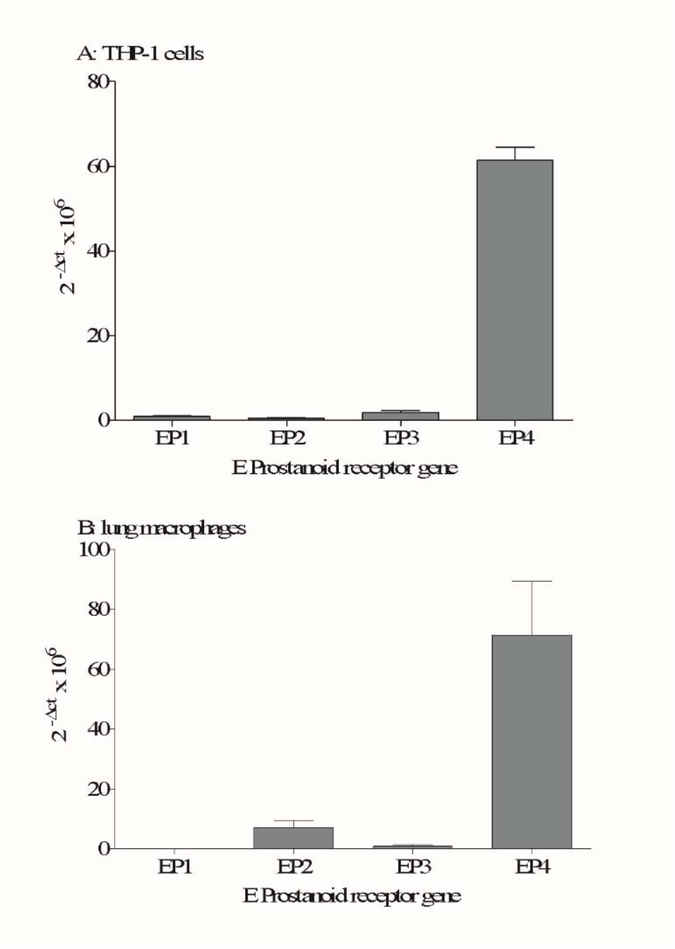

Supplement: Web figures [file thoraxjnl-2014-206592-s1.pdf]
